# Predictors of Multiwave Opioid Use Among Older American Adults

**DOI:** 10.1093/geroni/igad068

**Published:** 2023-07-13

**Authors:** Gillian Fennell, Mireille Jacobson, Hanna Grol-Prokopczyk

**Affiliations:** Leonard Davis School of Gerontology, University of Southern California, Los Angeles, California, USA; Leonard Davis School of Gerontology, University of Southern California, Los Angeles, California, USA; Schaeffer Center for Health Policy & Economics, University of Southern California, Los Angeles, California, USA; Department of Sociology, University at Buffalo, SUNY, Buffalo, New York, USA

**Keywords:** Acute opioid use, CDC guidelines, Chronic pain, Doctor visits, Long-term opioid therapy, Lung disease, Region

## Abstract

**Background and Objectives:**

Despite limited analgesic benefits, long-term opioid therapy (L-TOT) is common among older adults with chronic pain. Extended opioid use poses a threat to older adults as aging metabolisms retain opioids for longer, increasing the risk of injury, overdose, and other negative health outcomes. In contrast to predictors of general opioid use, predictors of L-TOT in older adults are not well documented. We aimed to identify such predictors using all available data on self-reported opioid use in the Health and Retirement Study.

**Research Design and Methods:**

Using 5 waves of data, respondents (*N* = 10,713) aged 51 and older were identified as reporting no opioid use (*n* = 8,621), a single wave of use (*n* = 1,410), or multiple waves of use (*n* = 682). We conducted a multinomial logistic regression to predict both single- and multiwave opioid use relative to no use. Demographic, socioeconomic, geographic, health, and health care–related factors were included in our model.

**Results:**

Multivariable findings show that, relative to nonusers, both single- and multiwave users were significantly more likely to be younger (relative risk ratio [RRR] = 1.33; RRR = 2.88); report lower household wealth (RRR = 1.47; RRR = 2.88); live in the U.S. Midwest (RRR = 1.29; RRR = 1.56), South (RRR = 1.34; RRR = 1.58), or West (RRR = 1.46; RRR = 2.34); experience interfering pain (RRR = 1.59; RRR = 3.39), back pain (RRR = 1.35; RRR = 1.53), or arthritic pain (RRR = 1.46; RRR = 2.32); and see the doctor frequently (RRR = 1.50; RRR = 2.02). Multiwave users were less likely to be Black (RRR = 0.69) or Hispanic (RRR = 0.45), and less likely to be never married (RRR = 0.52).

**Discussion and Implications:**

We identified demographic, socioeconomic, geographic, and health care-related predictors of chronic multiyear opioid use. Our focus on individuals taking opioids for this extended duration is novel. Differences in opioid use by geographic region and frequency of doctor visits particularly warrant attention from policy-makers and researchers. We make additional recommendations based on a sensitivity analysis limited to 2016–2020 data.

**Translational Significance:** We identify predictors of multiyear opioid use among older adults to inspire avenues of intervention for policy-makers. Opioid use for chronic pain typically provides limited pain relief and contributes to negative health outcomes, especially for older people. Predictors of multiyear use include younger age, non-Hispanic White ethnicity, low wealth, residing outside of the Northeast, pain, and frequent doctor visits. Lung disease significantly predicted multiyear use in the 2016–2020 model. Clinicians should remain cautious in their prescribing practices, especially for working-age patients who return frequently and have lung disease. Policy-makers should investigate contributors to regional patterns in opioid use.

## Background and Objectives

Although recent public health recommendations generally discourage opioid prescription, and randomized controlled trials demonstrate that, in many cases, opioids provide no more pain relief than over-the-counter nonopioid analgesics, opioids remain a common treatment for pain (Abdel Shaheed et al., 2016; Chang et al., 2017; Dowell et al., 2016; Myers et al., 2014; Thorlund et al., 2022). Opioid prescription rates have decreased since the mid-2000s, particularly after a widely publicized 2016 Centers for Disease Control and Prevention (CDC) recommendation to reduce opioid prescription (Asfaw et al., 2020; Dowell et al., 2016; Pensa et al., 2018), but this decrease has been gradual and partial (Bohnert et al., 2018). Indeed, given the consistent rise in U.S. pain prevalence (Zajacova et al., 2021), physicians continue to prescribe opioids, and not only for acute pain. In 2018, 15% of Americans were prescribed opioid medications for pain, both acute and chronic (Roehler et al., 2019).

Use of opioids for chronic pain may have unintended consequences. Long-term opioid use (or, interchangeably, long-term opioid therapy [L-TOT])—characterized by consistent use lasting longer than 90 days (Dowell et al., 2016)—now describes prescribed use patterns for 5%–12.5% of Americans with moderate-to-severe chronic pain (Clarke et al., 2014; Durand et al., 2019; Grol-Prokopczyk, 2019; Reuben et al., 2015; Shah et al., 2017). Unfortunately, when opioids are taken for this length of time, individuals tend to develop tolerance (Hayhurst & Durieux, 2016), requiring larger doses and making it more likely that analgesic benefits will be outweighed by significant health costs. Individuals risk the possibility of overdose, further injury (e.g., bone fractures), and myocardial infarction (Chou et al., 2015). Long-term opioid therapy also increases the likelihood of unemployment and higher health care costs (Eriksen et al., 2006; Johnston et al., 2016). For these reasons, L-TOT has received increasing attention and is specifically addressed in the 2022 update of the CDC’s recommendation for opioid prescription. In particular, the agency recommends that physicians seek opportunities to taper patients off of opioids or otherwise mitigate the risks of L-TOT whenever possible (Dowell et al., 2022).

Although concerns about opioids’ harms have focused largely on adults in midlife, older adults constitute a disproportionate share of long-term opioid users. This may stem in part from the relationship between age and chronic pain, which afflicts 31% of adults aged 65 and older compared to 8.5% of 18–29 years olds (Zelaya et al., 2020). As a consequence, older adults are also prescribed long-term opioids for chronic pain at a higher rate than younger groups: one study found that 16% of respondents with chronic pain aged 18–29 engaged in L-TOT compared to 64% of their older (65+) counterparts (Weissman et al., 2022). This high level of prescribing among older adults poses a problem as aging metabolisms make prescribing healthy and adequate doses of opioid medications more difficult (Chau, 2008; Tegeder et al., 1999). Opioids remain in the body for longer in older adults, and the consequences of long-term opioid use, including confusion, falls, bone fractures, nausea, constipation, urine retention, respiratory depression, increased pain sensitivity, and overdose, are amplified (Dunn, 2010; Ensrud et al., 2002; Furlan, 2006; Tilly et al., 2017).

Ample literature documents the predictors of general opioid use, which include low levels of education and wealth, disability, rural residence, identifying as White, having a blue-collar occupation, and advanced age (Armour et al., 2021; Asfaw et al., 2020; Grol-Prokopczyk, 2019; Heins et al., 2018; Pensa et al., 2018; Salans et al., 2022). However, predictors of *long-term* opioid use, especially among older adults, have received less attention.

The present study aims to identify predictors of long-term opioid use among adults over age 50. To do so, we exploit multiwave data on self-reported opioid use in the Health and Retirement Study (HRS). Updated understanding of these predictors is needed as clinical attitudes towards opioids and prescription rates have shifted greatly in the past decade, contributing to a possible shift in the type and importance of individual predictors of opioid use (Bloschichak & Hargraves, 2019; Dowell et al., 2016). Our findings may also identify vulnerable demographic, geographic, health-related, or socioeconomic subpopulations that could benefit from targeted personalized tapering from opioids and/or alternative, nonopioid treatment for pain.

## Research Design and Methods

### Data

We use data from the HRS, a longitudinal, nationally representative survey of Americans aged 51 and older (HRS, 2022a). The HRS is sponsored by the National Institute on Aging (grant number NIA U01AG009740) and is conducted by the University of Michigan. Our sample is restricted to respondents who were enrolled in the 2004 core survey and were asked about prescription drug use or any opioid use in subsequent survey waves. Specifically, data on opioid use came from the HRS Prescription Drug Studies (PDS) in 2005 and 2007 (HRS, 2008, 2011) and/or the three most recent biennial core waves in 2016, 2018, and 2020.

Prior to the introduction of a question about self-reported opioid use in the 2016 core survey, the two PDS surveys were administered on a subsample of the 2004 and 2006 HRS core samples in 2005 and 2007, respectively. Information about the sample selection for these studies is detailed in HRS documentation (HRS, 2008, 2011). General eligibility requirements for the 2005 PDS include being born in 1942 or earlier (age 63 or older) and not being selected for the Consumption and Activities Mail Survey, which was administered during the same time frame. The 2005 PDS oversampled individuals without insurance coverage for prescription drugs and/or those reporting low income and wealth. Respondents to the 2007 PDS included all individuals in the initial sample of the 2005 PDS who either completed the 2005 survey or were interviewed during the 2006 core wave.

In total, we use data from the cross-wave tracker file (HRS, 2022b), five core waves (2004, 2006, 2016, 2018, and 2020), and the two PDS (2005 and 2007). Additionally, we integrated cleaned HRS data from the RAND Corporation that corresponds to each of the core interview waves (RAND, 2022), as well as a household wealth variable from RAND’s detailed imputation files (RAND, 2021).

There were 14,370 cohort-eligible, community-dwelling respondents interviewed in 2004, 2006, 2016, 2018, and/or 2020 waves. Respondents were not excluded based on whether they were using opioids, allowing us to examine individuals reporting no opioid use, single-wave use, and multiwave use. Single-wave users were required to have been interviewed for at least one other wave, thus improving our confidence that they were not “multiwave users.” This requirement accounted for 2,367 excluded respondents. Because of the potentially important role of occupation for pain and subsequent opioid use, we used multiple imputation to address missing values for longest-held occupation. Multiple imputation procedures are outlined in the Independent Variables section. We excluded respondents missing data on our other variables of interest, who totaled less than 5% of the sample. In particular, we lost respondents due to missing values on variables indicating the number of doctor visits since the last wave (*n* = 374), household wealth (*n* = 292), urbanicity (*n* = 284), occupation (*n* = 155), health care satisfaction (*n* = 146), pain interference (*n* = 24), back pain (*n* = 7), marital status (*n* = 4), insurance status (*n* = 3), and education (*n* = 1). The final analytic sample consisted of 10,713 respondents.

### Outcome Measure

In both the 2005 and 2007 PDS, respondents were asked to write down the names of the prescription medications they were currently taking. We identified respondents who were taking opioids using a complete reference list of opioid drug names from John Hopkins Medicine (Butanis, 2018). In the core waves 2016–2020, the survey provided a description and examples of opioids, and then asked if the respondent had “taken any opioid pain medications in the past three months?” Our outcome opioid use measure is an indicator of no reported opioid use (0), single-wave opioid use (1; reported opioid use in just one wave), and multiwave opioid use (2; reported use in two or more waves). We did not require opioid use to be reported in consecutive waves to be considered “multiwave use” in the primary analysis. We also conducted an analysis with nonusers, single-wave users, and *consecutive* multiwave users to ensure our results were not sensitive to this decision; they were not.

### Independent Variables

The predictors we test in our analysis include sociodemographic, socioeconomic, geographic, health, and health care-related characteristics from 2004. Sociodemographic predictors include age (50–59, 60–69, 70+), gender, race/ethnicity (Non-Hispanic White, Non-Hispanic Black, Non-Hispanic Other, Hispanic), and marital status (married/partnered, separated/divorced, widowed, never married). Socioeconomic characteristics include educational attainment (less than high school, high school, college degree, or more), quartiles of household wealth (with the fourth quartile representing the highest wealth category), and longest-held occupation (professional/managerial, sales/clerical, service, and manual/operators). Geographical predictors included indicators for urbanicity (urban, suburban, or rural) and respondents’ U.S. region of residence using broad Census Bureau categories (Northeast, Midwest, South, or West). Seven health conditions were adjusted for past or present cancer, lung disease, heart disease, stroke, diabetes, arthritis, and high blood pressure. Health variables also included indicators for back pain, pain-related interference with daily activities, and difficulty with one or more activities of daily living (i.e., bathing, toileting, dressing, etc.), or instrumental activity of daily living (i.e., using the phone, preparing meals, managing money, etc.), respectively. Lastly, health care-related characteristics were measured through respondents’ number of doctor visits since the last wave (excluding hospital stays, outpatient surgeries, physical therapy appointments, and rehabilitation services; coded as 0–4, 5–10, and 11+ visits) and their general care satisfaction (satisfied, neutral, dissatisfied). For insurance type, respondents were categorized into (0) uninsured, (1) any private insurance, and (2) only public insurance.

To address the high proportion of missing values on the variables indicating longest-held occupation in 2004 (*n* = 1,846, 15.3%), we conducted multiple imputations using chained equations (Azur et al., 2011). To accommodate the categorical nature of longest-held occupation, we used multinomial logistic regression for imputation procedures. We estimated 10 iterations of imputed values for longest-held occupation using age, gender, race, marital status, educational attainment, income, wealth, region, urbanicity, and presence of activities of daily livings (ADL(s)) and instrumental activities of daily livings (IADL(s)). Although this technique successfully imputed values for the majority of the missing data on this variable, some missingness remained (*n* = 157).

### Analysis

We began by testing for multicollinearity between our variables of interest using a variance inflation factor (VIF) test. All predictors had VIF values lower than the well-used threshold of 5.0 (Studenmund & Johnson, 2017), indicating no strong multicollinearity. Next, we described the sample characteristics in aggregate and by opioid use and duration. To estimate the relative risk ratio (RRR) of reporting single-wave or multiwave opioid use relative to no use in our sample, we conducted a multinomial logistic regression including all of the sociodemographic, socioeconomic, geographic, health, and health care-related predictors described in the prior section. This analysis was pooled over the 10 imputed data sets for our mi set variable.

In addition to this primary analysis, we conducted three sensitivity checks to further aid our interpretation of the data. The first limited the analytic sample to only respondents aged 63 and older to assess whether findings would remain consistent if we conformed to the age eligibility criteria of the 2005 and 2007 PDS surveys. The second sensitivity check assessed only opioid use patterns documented in the most recent core waves (2016, 2018, and 2020), as predictors of general and long-term opioid use may have evolved following the recognition of the opioid epidemic and the firm CDC recommendation against unnecessary opioid prescription in 2016. Lastly, the third sensitivity test limited the sample to respondents reporting any pain in 2004 in order to separately identify factors that increase the risk of single- and multiwave opioid use from factors that predict pain.

For the second sensitivity check, we imputed 2016 longest-held occupation, number of doctor visits, and wealth to aid excessive missingness (>5% of the sample). Ten new imputed data sets were generated based on a similar set of characteristics as in the previously described imputation, except using 2016 values; this set also includes pain interference, health care satisfaction, seven chronic conditions, and enrollment in Medicare and Medicaid. This approach reduced missingness on all three variables, although relatively large proportions of missing values still remained: proportions of missingness for occupation, doctor visits, and wealth were 18.3%, 20.8%, and 21.2%, respectively.

All models were conducted in Stata 17 and weighted to generate estimates that were representative of the U.S. population. Respondent-level weights correct for nonresponse bias, complex survey design, and poststratification adjustments. For example, they adjust for the survey’s oversampling of African Americans, Hispanics, and Floridians and account for the different sampling frame used for “oldest old” respondents (Willis et al., 2006).

## Results

Table 1 documents characteristics of the total sample (*N* = 10,713) and stratifies them by opioid use pattern. Single-wave and multiwave opioid users make up 13.2% and 6.3% of the total sample, respectively. The full sample of individuals was majority White (81.4%) and majority females (56.0%). Nearly half (44.2%) of the sample were between the ages of 51 and 59, and 25.2% were aged 70 or older. In terms of health, the total sample reported distress, pain, and associated outcomes at the following rates: depression (22.8%), back pain (36.9%), arthritis (48.0%), interfering pain (18.3%), difficulty with one or more ADLs (9.7%), and difficulty with one or more IADLs (8.4%).

**Table 1.** HRS 2004 Baseline Characteristics in Aggregate and Stratified By 2004–2020 Opioid Use Patterns (*N* = 10,713)

Characteristic	Total sample (*N* = 10,713)	No reported opioid use (*n* = 8,621)	Single-wave opioid use (*n* = 1,410; 13.2%)		Multiwave opioid use (*n* = 682; 6.3%)	
	%	%	%		%	
*Demographics*						
Female	56.0	55.4	57.2		60.9	^*^
Race						
Non-Hispanic White (ref)	81.4	81.4	80.7		83.0	
Non-Hispanic Black	8.8	8.7	9.5		8.4	
Non-Hispanic Other	2.7	2.5	3.8	^*^	3.6	
Hispanic	7.1	7.5	6.0		5.0	^*^
Age						
51–59	44.2	43.0	45.7	^†^	55.3	^***^
60–69	30.5	30.7	30.2		29.0	^***^
70+ (ref)	25.2	26.3	24.1		15.7	
Marital status						
Married/partnered (ref)	67.8	68.7	64.7		64.3	
Separated/divorced	14.8	13.9	17.8	^**^	19.1	^**^
Widowed	13.6	13.5	13.8		14.1	
Never married	3.7	3.9	3.7		2.4	
*Socioeconomic status*						
Education						
Less than high school (ref)	19.4	19.1	19.8		22.1	
High school	29.8	30.2	27.1		30.1	
College and above	50.8	50.7	53.1		47.8	^†^
Wealth						
1 (lowest)	17.1	15.8	21.3	^***^	23.1	^***^
2	24.7	24.2	25.4	^*^	29.0	^***^
3	28.8	29.1	27.6		28.2	^**^
4 (highest; ref)	29.4	30.9	25.7		19.7	
Occupation						
Managerial (ref)	33.5	33.7	34.8		29.0	
Sales/clerical	27.9	28.1	27.5		27.0	
Service	13.6	13.3	12.9		17.8	^**^
Manual/operators	24.9	24.8	24.8		26.1	
*Geography*						
Beale rural–urban code						
Urban (ref)	49.6	49.8	49.5		47.9	
Suburban	22.4	22.6	20.9		23.1	
Rural	27.9	27.6	29.6		29.0	
Region						
Northeast (ref)	17.0	18.0	13.9		11.2	
Midwest	26.6	26.7	26.4	^*^	25.7	^**^
South	36.3	35.9	38.0	^**^	37.2	^**^
West	20.2	19.4	21.6	^**^	25.9	^***^
*Health conditions*						
Depression	22.8	21.4	25.5	^**^	33.6	^***^
Back pain	36.9	32.7	47.5	^***^	62.9	^***^
Arthritis	48.0	43.8	58.6	^***^	73.8	^***^
Pain interference						
No pain (ref)	68.4	73.5	57.3		33.2	
Noninterfering pain	13.2	12.3	15.5	^***^	18.6	^***^
Interfering pain	18.3	14.1	27.2	^***^	48.2	^***^
ADL	9.7	8.0	13.2	^***^	21.4	^***^
IADL	8.4	7.3	11.3	^***^	15.5	^***^
Ever had cancer	9.6	9.4	10.5		9.7	
Lung disease	5.3	4.8	7.3	^**^	7.5	^*^
Heart disease	15.5	14.9	18.5	^**^	16.8	
Stroke	4.3	3.9	5.8	^**^	5.8	^†^
Diabetes	13.1	12.4	15.9	^**^	15.6	^†^
High blood pressure	44.5	43.0	50.2	^***^	49.6	^**^
*Health care-related characteristics*						
Care satisfaction						
Neutral (ref)	39.8	39.1	41.8		43.6	
Satisfied	52.8	53.8	51.1		45.5	^***^
Dissatisfied	7.4	7.1	7.1		10.9	^†^
# Times seen a doctor						
0–4 times (ref)	44.5	47.5	35.6		28.5	
5–10 times	33.7	33.5	35.6	^***^	32.0	^***^
11+ times	21.8	19.1	28.8	^***^	39.5	^***^
Insurance status						
Uninsured (ref)	8.0	8.0	8.1		7.9	
Any private	75.2	76.0	73.1		71.3	
Public only	16.8	16.1	18.9		20.8	

*Notes*: ADL = activities of daily living; HRS = Health and Retirement Study; IADL = instrumental activities of daily living. Significance testing is relative to respondents who reported no opioid use throughout the study period.

^†^ *p* < .10.

^*^ *p* < .05.

^**^ *p* < .01.

^***^ *p* < .001.

Relative to nonopioid users, single-wave users were more likely to be divorced or separated, have lower household wealth, live in the South or West, have depression, and report activity-interfering, arthritic, and back pain. Single-wave users were also more likely to suffer from six of the seven chronic health conditions (excluding cancer), report difficulty with ADLs and IADLs, and make more frequent visits to the doctor. Multiwave users also share the same regional, wealth, and functional patterning as single-wave users. Relative to nonopioid users, multiwave users were more likely to be younger, female, and separated/divorced, respectively. Additionally, multiwave users were more likely to work service jobs, less likely to identify as Hispanic, and less likely to be satisfied with their health care than nonopioid users.

Table 2 showcases RRR results from the multinomial logistic regression simultaneously assessing all predictors of single- and multiwave opioid use. The associations between each predictor and opioid use pattern should be interpreted net of all other covariates and relative to no use. We focus here primarily on predictors of multiwave use, although findings regarding single-wave opioid use are also shown in Table 2 (and significant predictors for both are often similar). Younger respondents were more likely to report multiwave opioid use (50–59: RRR = 2.88, *p* < .001; 60–69: RRR = 1.76, *p* < .001), whereas those who were never married reported a lower risk (RRR = 0.52, *p* < .05). Relative to Non-Hispanic Whites, respondents identifying as Non-Hispanic Black (RRR = 0.69, *p* < .05) or Hispanic (RRR = 0.44, *p* < .001) were significantly less likely to report multiwave use. Individuals with lower wealth (Q1: RRR = 1.55, *p* < .05; Q2: RRR = 1.52, *p* < .01), and those residing outside of the Northeast were more likely to be multiwave users: Midwest (RRR = 1.56, *p* < .05), South (RRR = 1.58, *p* < .01), and West (RRR = 2.34, *p* < .001). Lastly, pain sufferers (interfering pain: RRR = 3.39, *p* < .001; back pain: RRR = 1.53, *p* < .001; arthritis: RRR = 2.32, *p* < .001) and individuals who visit the doctor frequently (5–10 times: RRR = 1.30, *p* < .05; 11+ times: RRR = 2.02, *p* < .001) were at higher risk for reporting multiwave opioid use.

**Table 2.** Multinomial Logistic Regression Using 2004 Covariates to Predict Single- and Multiwave Opioid Use in 2004–2020 (*N* = 10,713)

Characteristic	Single-wave opioid use			Multiwave opioid use		
	RRR		SE	RRR		SE
*Demographics*						
Female	0.94		0.08	0.91		0.11
Race						
White (ref)						
Black	0.91		0.10	0.69	^*^	0.12
Other	1.37		0.29	1.06		0.10
Hispanic	0.76	^†^	0.11	0.45	^***^	0.24
Age						
51–59	1.33	^**^	0.13	2.88	^***^	0.43
60–69	1.14		0.10	1.76	^***^	0.24
70+ (ref)						
Marital status						
Married/partnered (ref)						
Separated/divorced	1.19	^†^	0.12	1.09		0.16
Widowed	1.04		0.11	1.27		0.22
Never married	0.93		0.19	0.52	^*^	0.17
*Socioeconomic status*						
Education						
Less than high school (ref)						
High school	0.96		0.10	1.00		0.15
College and above	1.22	^†^	0.13	1.12		0.19
Wealth						
1 (lowest)	1.47	^**^	0.18	1.55	^*^	0.29
2	1.23	^*^	0.13	1.52	^**^	0.23
3	1.12		0.11	1.32	^†^	0.19
4 (highest; ref)						
Occupation						
Managerial (ref)						
Sales/clerical	0.95		0.09	0.99		0.14
Service	0.87		0.11	1.27		0.24
Manual/operators	0.89		0.10	0.97		0.17
*Geography*						
Beale rural–urban code						
Urban (ref)						
Suburban	0.89		0.08	0.98		0.12
Rural	1.04		0.09	0.93		0.12
Region						
Northeast (ref)						
Midwest	1.29	^*^	0.15	1.56	^*^	0.27
South	1.34	^**^	0.15	1.58	^**^	0.26
West	1.46	^**^	0.18	2.34	^***^	0.40
*Health conditions*						
Depression	0.93		0.08	1.00		0.12
Back pain	1.35	^***^	0.11	1.53	^***^	0.18
Arthritis	1.46	^***^	0.12	2.32	^***^	0.30
Pain interference						
No pain (ref)						
Noninterfering pain	1.31	^**^	0.14	2.23	^***^	0.34
Interfering pain	1.59	^***^	0.16	3.39	^***^	0.47
ADL	0.98		0.11	1.14		0.16
IADL	1.04		0.13	0.94		0.15
Ever had cancer	1.02		0.11	0.97		0.16
Lung disease	1.15		0.16	0.89		0.19
Heart disease	1.03		0.09	0.85		0.12
Stroke	1.18		0.17	1.10		0.24
Diabetes	1.07		0.11	0.93		0.13
High blood pressure	1.16	^*^	0.09	1.11		0.12
*Health care-related characteristics*						
Care satisfaction						
Neutral (ref)						
Satisfied	0.91		0.07	0.82	^†^	0.09
Dissatisfied	0.82		0.12	1.07		0.20
#Times seen a doctor						
0–4 times (ref)						
5–10 times	1.24	^*^	0.10	1.30	^*^	0.17
11+ times	1.50	^***^	0.14	2.02	^***^	0.28
Insurance status						
Uninsured (ref)						
Any private	1.01		0.15	1.31		0.28
Public only	1.00		0.16	1.29		0.30
*Constant*	0.10	^***^	0.02	0.02	^***^	0.01

*Note*: ADL = activities of daily living; IADL = instrumental activities of daily living; RRR = relative risk ratio; SE = standard error. Base outcome is no reported opioid use.

^†^ *p* < .10.

^*^ *p* < .05.

^**^ *p* < .01.

^***^ *p* < .001.

We conducted three sensitivity checks that contribute to our understanding of factors predicting opioid use patterns in an older sample, in more recent years, and among only individuals with pain, respectively. Results from these models are displayed in Table 3, and Supplementary Tables 1 and 2. When we restricted the sample to respondents aged 63 or older to match PDS eligibility (*n* = 6,365; Supplementary Table 1), the model yielded similar findings to the primary analysis, with both single- and multiwave use declining with age, and increasing with back pain, arthritis, and either noninterfering or interfering pain. Black respondents were less likely to identify as multiwave users. Differences relative to the full sample were mostly found in the factors predicting multiwave use—in this older sample, Hispanic identity, being never married, low wealth, living in the South or Midwest, and reporting frequent doctor visits were no longer significant predictors.

**Table 3.** Multinomial Logistic Regression Using 2016 Covariates to Predict Single- and Multiwave Opioid Use in 2016–2020 (*N* = 17,067)

Characteristic	Single-wave opioid use			Multiwave opioid use		
	RRR		SE	RRR		SE
*Demographics*						
Female	0.90		0.07	0.83		0.09
Race						
White (ref)						
Black	1.01		0.09	1.13		0.14
Other	0.90		0.13	0.84		0.16
Hispanic	0.77	^*^	0.09	0.56	^***^	0.09
Age						
51*–*59	1.45	^***^	0.13	2.32	^***^	0.29
60*–*69	1.32	^**^	0.10	1.64	^***^	0.18
70+ (ref)						
Marital status						
Married/partnered (ref)						
Separated/divorced	1.21	^*^	0.10	1.04		0.12
Widowed	0.94		0.08	0.90		0.11
Never married	0.92		0.14	0.69	^†^	0.14
*Socioeconomic status*						
Education						
Less than high school (ref)						
High school	0.87		0.08	1.14		0.14
College and above	1.05		0.11	1.19		0.17
Wealth						
1 (lowest)	1.24	^*^	0.14	1.09		0.16
2	1.30	^*^	0.13	1.07		0.15
3	1.30	^**^	0.13	1.03		0.14
4 (highest; ref)						
Occupation						
Managerial (ref)						
Sales/clerical	0.95		0.10	1.04		0.17
Service	0.92		0.13	1.07		0.18
Manual/operators	0.83		0.11	0.91		0.16
*Geography*						
Beale rural–urban code						
Urban (ref)						
Suburban	0.88		0.07	1.07		0.11
Rural	0.90		0.07	1.16		0.12
Region						
Northeast (ref)						
Midwest	1.21		0.13	1.30		0.21
South	1.36	^**^	0.14	1.51	^**^	0.22
West	1.45	^**^	0.16	1.98	^***^	0.31
*Health conditions*						
Depression	1.01		0.08	1.06		0.10
Back pain	1.52	^***^	0.11	2.38	^***^	0.25
Arthritis	1.48	^***^	0.11	2.28	^***^	0.27
Pain interference						
No pain (ref)						
Noninterfering pain	1.44	^***^	0.13	3.04	^***^	0.43
Interfering pain	1.97	^***^	0.17	6.65	^***^	0.83
ADL	1.04		0.10	1.28	^*^	0.14
IADL	1.24	^*^	0.12	1.22	^†^	0.14
Ever had cancer	1.09		0.09	0.92		0.10
Lung disease	1.26	^*^	0.12	1.32	^*^	0.15
Heart disease	1.02		0.08	1.06		0.10
Stroke	1.04		0.11	0.99		0.13
Diabetes	1.23	^**^	0.09	1.11		0.11
High blood pressure	1.07		0.08	1.07		0.11
*Health care-related characteristics*						
Care satisfaction						
Neutral (ref)						
Satisfied	0.82	^†^	0.08	0.93		0.12
Dissatisfied	0.79		0.12	1.04		0.18
# Times seen a doctor						
0–4 times (ref)						
5–10 times	1.34	^***^	0.11	1.25	^†^	0.15
11+ times	1.81	^***^	0.16	2.69	^***^	0.32
Insurance						
Uninsured (ref)						
Any private insurance	1.21		0.16	1.03		0.19
Only public insurance	1.28	^†^	0.17	1.13		0.22
*Constant*	0.04	^***^	0.01	0.00	^***^	0.00

*Note*: ADL = activities of daily living; IADL = instrumental activities of daily living; RRR = relative risk ratio; SE = standard error. Base outcome is no reported opioid use.

^†^ *p* < .10.

^*^ *p* < .05.

^**^ *p* < .01.

^***^ *p* < .001.

When we replicated our analysis on only the three most recent waves of HRS data (*n* = 17,067; Table 3), several differences with the primary analysis emerged. Identifying as Black was no longer significantly associated with single- or multiwave use. Household wealth, being never married, or living in the Midwest were also no longer significant predictors of multiwave use. The positive relationships between opioid use and the three measured pain variables (back pain, arthritis, noninterfering, and interfering pain) remained. In the later waves, having difficulty with IADLs, diabetes, and being separated/divorced were positively associated with single-wave opioid use, and reporting lung disease and ADL difficulties were positively associated with *both* single- and multiwave use.

Lastly, when we restricted the primary analytic sample to only include people reporting pain in 2004 (*N* = 3,363; Supplementary Table 2), observed patterns were generally similar to those for the overall sample but tended to be less precise (as expected given the smaller sample size). For example, we found that the RRRs by race/ethnicity, age, and pain-related covariates were similar in magnitude to the main sample estimates but only significantly associated with multiwave use. Wealth remained a significant predictor, but only for single-wave use. Predictive patterns by region and the number of doctor visits for both single- and multiwave use were similar to those in the primary model. Interestingly, in this model, manual workers and operators were 31% less likely to be single-wave users relative to individuals whose longest-held occupations were managerial in nature. Overall, these supplementary findings reassure us that our primary model identifies predictors of opioid use, not merely predictors of pain.

## Discussion and Implications

In the present study, we identify demographic, socioeconomic, geographic, and health-related predictors of single- and multiwave opioid use in the HRS.

### Demographic Predictors

Of the demographic variables, age, race, and marital status significantly predicted opioid use for some duration. Younger respondents (aged 51–59 and 60–69) were more likely to report either single- or multiwave use. Whereas previous studies have often concluded that *older* age predicts higher likelihood of opioid use, many of these studies treat “65+” as a single age group (Asfaw et al., 2020; Schieber et al., 2019). We disaggregate older age groups more finely, revealing lower rates of opioid use among those aged 70+. Given that the consequences of opioid use, and particularly long-term use, are amplified among older adults (e.g., falls, bone fracture, and confusion; Tilly et al., 2017; Yoshikawa et al., 2020), clinicians may be prescribing opioids less readily once patients age into their 70s and 80s. This finding may also be partially explained by survival selection bias: older individuals with pain severe enough to warrant opioid use may be less represented in our sample due to death or refusal to participate (Tolonen et al., 2017; Vartiainen et al., 2022).

The lower likelihood of long-term opioid use for Black and Hispanic respondents conforms with an abundant literature documenting discriminatory medical practices (Fiscella et al., 2000), albeit practices that may have inadvertently limited the oversupply of prescription opioids experienced in many White communities. Black and Hispanic patients have historically received lower doses of analgesics for pain of the same intensity as White counterparts (Dowell et al., 2022; Goyal et al., 2015; Hausmann et al., 2013; Lee et al., 2019; Morden et al., 2021). Additionally, if Black patients *were* prescribed opioids, clinicians were significantly more likely to stop writing prescriptions in response to signs of misuse relative to Whites (Gaither et al., 2018). In the 2016–2020 model, the negative relationship between Black identity and opioid use was no longer observed. This signals potentially positive progress against medical discrimination in recent years, but with potentially insidious consequences for older Black Americans: the 2020 overdose death rate for Black males over age 65 was seven times higher than that of White males in the same age group (Centers for Disease Control and Prevention, 2022; although illicit rather than licit opioids played a substantial role in this excess mortality).

With respect to marital status, our finding that respondents who were never married were less likely to be multiwave users was unexpected and not supported by prior work. The majority of research on the relationship between marital status and opioid use focuses on misuse and abuse: being single has been linked to opioid dependence and abuse (Edlund et al., 2007), and being married has been associated with a lower likelihood of misuse (Cragg et al., 2019). Consistent with this research, results from the 2016–2020 model suggest that being separated/divorced is associated with higher risk of single-wave opioid use. Future research would be needed to understand why being never married predicted lower multiwave use.

### Socioeconomic Predictors

Previous work has suggested that low levels of educational attainment, low wealth, and working blue-collar occupations are positive predictors of opioid use (Armour et al., 2021; Asfaw et al., 2020; Grol-Prokopczyk, 2019). We did not find the same educational and occupational patterns in our study, but our primary analysis supports the established negative relationship between wealth and any opioid use. Using HRS PDS data, Grol-Prokopczyk (2019) also showed that wealth significantly and negatively predicted opioid use, whereas education and occupation did not (within the same model). Similarly, our findings suggest that household wealth is a more important socioeconomic predictor of opioid use relative to education or occupation, at least in the early-to-mid 2000s. In our 2016–2020 model, low wealth was only predictive of single-wave use. Current literature does not sufficiently explain why wealth is no longer a predictor of L-TOT in recent years. Studies are beginning to test explanations for increased use of illicit opioids in the middle class (Grossmann & Strulik, 2021), but future research should continue to investigate new developments in the socioeconomic factors predicting acute and long-term opioid use.

### Geographic Predictors

In both the primary analysis and 2016–2020 model, living in the Midwest, South, or West relative to the Northeast increased the risk of both single- and multiwave opioid use. These findings are consistent with recent work documenting the same geographic variations in pain frequency, activity interference, and prescription opioid use (Zajacova et al., 2022, 2023). Schieber et al. (2019) also support our findings by showing that doctors practicing in Western and (predominantly) Southern states were prescribing opioids for 30 or more days significantly more frequently than other U.S. regions. Underlying reasons for these sometimes stark regional differences have not been elucidated, however.

### Health Predictors

Unsurprisingly, pain—whether it be arthritic pain, back pain, interfering pain, or noninterfering pain—was associated with higher risk of single- and multiwave opioid use across the primary and 2016–2020 models. These findings are consistent with prior work (Armour et al., 2021; Pensa et al., 2018).

We also found that having lung disease at baseline was associated with increased risk of opioid use in our 2016–2020 model. These medications have been found to relieve breathlessness in palliative care settings for individuals with chronic obstructive pulmonary disease (COPD) (Keogh & Williams, 2021). However, evidence showcasing the efficacy of opioids for this purpose is mixed and of low quality (Barnes et al., 2016; Chen et al., 2022; Verberkt et al., 2017). In fact, opioid use has been implicated in the exacerbation of COPD symptoms, especially at higher doses (Ramachandran et al., 2021). Although low doses of opioids for breathlessness do not increase risk of mortality (Chen et al., 2022; Ekstrom et al., 2014), clinicians should be cautious as the burden of opioid side effects (e.g., respiratory depression, nausea, and vomiting) may outweigh provided benefits.

We expected that depression would be a significant predictor of opioid use given the strong relationship between pain and depression (Dueñas et al., 2016; Goesling et al., 2013; Steiner et al., 2017; Vos et al., 2016). This was not confirmed; however, our nonsignificant findings do correspond with previous literature. A growing number of recent studies have found that although depression does not predict opioid prescription, larger doses and extended duration of opioid use do increase the risk of major depression (Feingold et al., 2017; Leung et al., 2022; Rosoff et al., 2021; Semenkovich et al., 2014; Sullivan, 2018). Our study builds on this literature by demonstrating that this relationship is not bidirectional in a nationally representative sample.

Activity of daily living difficulties positively predicted multiwave opioid use, and IADL difficulties predicted single-wave use in the 2016–2020 model despite neither being significant predictors in our primary analysis. These findings may signal that, following the 2016 CDC recommendation, clinicians may have stopped prescribing opioids to patients whose pain and pain-related disabilities were not severe (Dowell et al., 2016).

### Health Care-related Predictors

Of three health care-related factors (care satisfaction, frequency of doctor visits, and insurance type), only frequent doctor visits predicted single- and multiwave use. To our knowledge, this is the first study to test and detect such an association. This finding could reflect that individuals with pain and other health issues visit their doctor more frequently, thus increasing their likelihood to receive pharmacological treatment—including opioids. The association may also reflect “doctor-shopping,” whereby individuals make frequent visits to different doctors in pursuit of an eventual opioid prescription. Prior work has not explored such possibilities, so these reasons remain speculative for now; future research should continue to explore reasons for this association.

## Limitations

This study has several limitations. We used single-wave and multiwave opioid use as a proxy for acute and long-term opioid use as there is no way to precisely characterize the traditional “90 days or more” definition of L-TOT using the HRS. Our findings may thus be less applicable to all “long-term” users and more so to individuals using opioids for at least several years. However, considering that this subset of long-term users accounts for 6.3% of our total sample from a nationally representative data set, we still regard these findings as important to our understanding of L-TOT. In fact, these results may provide insight into a particularly vulnerable subset of long-term opioid users given their duration of use and advanced age.

Another potential limitation of our study is that the PDS respondents were asked about all prescription drug use (from which we identified prescription opioid use) whereas the 2016–2020 core respondents were asked about *any* opioid use, prescription or not. Although rates of opioid misuse have significantly increased among Americans aged 55+ over the last decade (Shoff et al., 2021), the vast majority of older adults who are misusing these medications are prescribed them (Cicero et al., 2012; Schepis et al., 2020). Therefore, the distinction between “prescribed” and “any” opioid use may not be meaningful in our sample. However, it is possible that we are identifying aggregated predictors for two distinct types of opioid use and thereby missing differences in the predictors of any one type of use.

We also acknowledge our use of covariates from 2004 to predict duration of opioid use through 2020 as a limitation. Variables that are not stable over time, like marital status and diagnosed chronic conditions, may have changed during the study period. Nonetheless, we used an early baseline for covariates to limit the potential for reverse causation. Changes in marital status, pain, and ADL difficulty over the study period may have occurred *because of* chronic opioid use and not the reverse. Importantly, our model assessing 2016–2020 opioid use that uses baseline covariates from 2016 improved our confidence in these earlier findings, as both identified a number of similar predictors (including ethnicity, Census region, pain, and frequency of seeing a doctor) as well as similar nonpredictors.

## Conclusion

Opioid prescribing has declined in recent years, but a substantial number of Americans continue to receive ongoing or long-term opioid prescriptions. We have identified five factors that play significant roles in predicting multiwave opioid use among older adults in both our 2004–2020 and 2016–2020 models: younger age (50–59), being non-Hispanic white, residing in either the South or West, experiencing pain, and frequent doctor visits. Significant predictive factors that were only detected in more recent years (2016–2020) include ADL limitations and lung disease. Identifying as Black, being never married, reporting low wealth, and living in the Midwest seemed to be meaningful factors that either increased or reduced risk of L-TOT earlier in the opioid epidemic, as shown in Table 2. These findings help identify populations that may need more appropriate, long-term pain management.

In light of our findings, policy-makers should explore what factors may be leading to sustained opioid use outside of the Northeastern region. To do this, future research should use more fine-grained geographic data, as our broad regional categories cannot adequately direct policy change at state or lower levels. Additionally, given our finding that individuals in the youngest age category (50–59) were at greatest risk of multiwave use, working-age Americans may be a specific target for nonopioid alternatives. By targeting this group before they reach more advanced ages, we may prevent people from continuing opioid therapies into old age, and thereby reduce unnecessary negative health outcomes in this population. This potential to reduce future L-TOT in older age groups is particularly salient as recent work has demonstrated that today’s middle-aged adults report more pain than today’s older adults did when they were a similar age (Case et al., 2020).

We believe this study is the first to identify frequent doctor visits as a predictor of opioid use. Future research should devote efforts to understand whether this finding signals that individuals are successfully advocating for their care needs or are taking advantage of the prescribing power of their clinicians. Clinicians should be aware of this association, as well as the associations between both lung disease and diabetes with opioid use, as they reflect upon and consider adjusting their opioid prescribing practices.

## Supplementary Material

igad068_suppl_Supplementary_Material
