# Supplementary material for: Predictors of Multiwave Opioid Use Among Older American Adults
Source: Innov Aging. 2023 Jul 13;7(10):igad068. doi: 10.1093/geroni/igad068 (PMC10714904; doi:10.1093/geroni/igad068)
Supplement: igad068_suppl_Supplementary_Material [file igad068_suppl_supplementary_material.docx]

**Online Supplementary Material**

**Table S1.** Multinomial logistic regression using 2004 covariates to predict single- and multi-wave opioid use among respondents aged 63+ in 2004-2020 (N = 6,365)

| **Characteristic** | **Single wave opioid use** | | |  | **Multi-wave opioid use** | | |
| --- | --- | --- | --- | --- | --- | --- | --- |
|  | ***RRR*** | | ***SE*** |  | ***RRR*** | | ***SE*** |
| *Demographics* |  |  |  |  |  |  |  |
| Female | 1.21 | † | 0.13 |  | 1.07 |  | 0.17 |
| Race |  |  |  |  |  |  |  |
| White (ref) |  |  |  |  |  |  |  |
| Black | 0.88 |  | 0.13 |  | 0.51 | ** | 0.12 |
| Other | 0.41 | * | 0.16 |  | 0.60 |  | 0.31 |
| Hispanic | 0.64 | * | 0.12 |  | 0.60 | † | 0.17 |
| Age | 0.98 | * | 0.01 |  | 0.96 | *** | 0.01 |
| Marital Status |  |  |  |  |  |  |  |
| Married/partnered (ref) |  |  |  |  |  |  |  |
| Separated/divorced | 1.15 |  | 0.17 |  | 1.01 |  | 0.21 |
| Widowed | 1.16 |  | 0.13 |  | 1.15 |  | 0.21 |
| Never married | 0.93 |  | 0.27 |  | 0.71 |  | 0.38 |
| *Socioeconomic status* |  |  |  |  |  |  |  |
| Education |  |  |  |  |  |  |  |
| Less than high school (ref) |  |  |  |  |  |  |  |
| High school | 0.95 |  | 0.11 |  | 0.97 |  | 0.18 |
| College and above | 1.19 |  | 0.16 |  | 1.17 |  | 0.24 |
| Wealth |  |  |  |  |  |  |  |
| 1 (lowest) | 1.41 | * | 0.22 |  | 1.03 |  | 0.24 |
| 2 | 1.02 |  | 0.14 |  | 1.15 |  | 0.22 |
| 3 | 1.21 |  | 0.14 |  | 1.01 |  | 0.18 |
| 4 (highest; ref) |  |  |  |  |  |  |  |
| Occupation |  |  |  |  |  |  |  |
| Managerial (ref) |  |  |  |  |  |  |  |
| Sales / Clerical | 0.85 |  | 0.10 |  | 1.11 |  | 0.25 |
| Service | 0.87 |  | 0.14 |  | 1.09 |  | 0.34 |
| Manual / Operators | 1.00 |  | 0.14 |  | 1.08 |  | 0.26 |
| *Geography* |  |  |  |  |  |  |  |
| Beale Rural - Urban code |  |  |  |  |  |  |  |
| Urban (ref) |  |  |  |  |  |  |  |
| Suburban | 1.00 |  | 0.11 |  | 1.09 |  | 0.17 |
| Rural | 1.03 |  | 0.11 |  | 0.84 |  | 0.14 |
| Region |  |  |  |  |  |  |  |
| Northeast (ref) |  |  |  |  |  |  |  |
| Midwest | 1.43 | * | 0.21 |  | 1.13 |  | 0.25 |
| South | 1.58 | ** | 0.22 |  | 1.26 |  | 0.26 |
| West | 1.70 | ** | 0.27 |  | 1.55 | * | 0.34 |
| *Health Conditions* |  |  |  |  |  |  |  |
| Depression | 0.98 |  | 0.10 |  | 1.06 |  | 0.17 |
| Back pain | 1.26 | * | 0.12 |  | 1.82 | *** | 0.27 |
| Arthritis | 1.52 | *** | 0.16 | i | 2.32 | *** | 0.41 |
| Pain interference |  |  |  |  |  |  |  |
| No pain (ref) |  |  |  | W |  |  |  |
| Non-Interfering pain | 1.74 | *** | 0.21 |  | 2.33 | *** | 0.45 |
| Interfering pain | 1.80 | *** | 0.22 |  | 3.95 | *** | 0.67 |
| ADL | 1.21 |  | 0.17 |  | 1.36 | † | 0.25 |
| IADL | 1.05 |  | 0.16 |  | 0.84 |  | 0.19 |
| Ever had cancer | 1.14 |  | 0.14 |  | 0.89 |  | 0.18 |
| Lung disease | 1.10 |  | 0.17 |  | 0.66 |  | 0.17 |
| Heart disease | 0.96 |  | 0.10 |  | 1.06 |  | 0.17 |
| Stroke | 1.08 |  | 0.17 |  | 0.77 |  | 0.22 |
| Diabetes | 0.87 |  | 0.10 |  | 0.86 |  | 0.16 |
| High blood pressure | 1.07 |  | 0.10 |  | 1.10 |  | 0.15 |
| *Healthcare related characteristics* |  |  |  |  |  |  |  |
| Care satisfaction |  |  |  |  |  |  |  |
| Neutral (ref) |  |  |  |  |  |  |  |
| Satisfied | 0.99 |  | 0.09 |  | 0.95 |  | 0.13 |
| Dissatisfied | 0.84 |  | 0.17 |  | 0.90 |  | 0.25 |
| # Times seen a doctor |  |  |  |  |  |  |  |
| 0-4 times (ref) |  |  |  |  |  |  |  |
| 5-10 times | 1.04 |  | 0.11 |  | 1.12 |  | 0.19 |
| 11+ times | 1.27 | * | 0.14 |  | 1.28 |  | 0.22 |
| Insurance status |  |  |  |  |  |  |  |
| Uninsured (ref) |  |  |  |  |  |  |  |
| Any private | 0.72 |  | 0.17 |  | 1.37 |  | 0.65 |
| Public only | 0.74 |  | 0.18 |  | 1.55 |  | 0.72 |
| *Constant* | 0.21 | ** | 0.11 |  | 0.14 | * | 0.17 |

*Note.* ADL = activities of daily living; IADL = instrumental activities of daily living. Base outcome is no reported opioid use.

† p<.10 * p<.05; ** p<.01; *** p<.001.

**Table S2.** Multinomial logistic regression using 2004 covariates to predict single- and multi-wave opioid use in 2004-2020 among respondents reporting pain in 2004 (N=3,363)

| **Characteristic** | **Single wave opioid use** | | |  | **Multi-wave opioid use** | | |
| --- | --- | --- | --- | --- | --- | --- | --- |
|  | ***RRR*** | | ***SE*** |  | ***RRR*** | | ***SE*** |
| *Demographics* |  |  |  |  |  |  |  |
| Female | 0.99 |  | 0.13 |  | 0.92 |  | 0.14 |
| Race |  |  |  |  |  |  |  |
| White (ref) |  |  |  |  |  |  |  |
| Black | 0.78 |  | 0.14 |  | 0.67 | † | 0.14 |
| Other | 1.49 |  | 0.46 |  | 1.12 |  | 0.43 |
| Hispanic | 0.82 |  | 0.17 |  | 0.43 | ** | 0.11 |
| Age |  |  |  |  |  |  |  |
| 51-59 | 1.12 |  | 0.17 |  | 2.55 | *** | 0.47 |
| 60-69 | 1.04 |  | 0.14 |  | 1.58 | ** | 0.27 |
| 70+ (ref) |  |  |  |  |  |  |  |
| Marital Status |  |  |  |  |  |  |  |
| Married/partnered (ref) |  |  |  |  |  |  |  |
| Separated/divorced | 1.10 |  | 0.18 |  | 1.17 |  | 0.20 |
| Widowed | 0.92 |  | 0.15 |  | 1.23 |  | 0.25 |
| Never married | 1.01 |  | 0.32 |  | 0.62 |  | 0.25 |
| *Socioeconomic status* |  |  |  |  |  |  |  |
| Education |  |  |  |  |  |  |  |
| Less than high school (ref) |  |  |  |  |  |  |  |
| High school | 0.92 |  | 0.14 |  | 0.95 |  | 0.17 |
| College and above | 1.16 |  | 0.20 |  | 1.19 |  | 0.24 |
| Wealth |  |  |  |  |  |  |  |
| 1 (lowest) | 1.96 | ** | 0.39 |  | 1.22 |  | 0.28 |
| 2 | 1.51 | * | 0.26 |  | 1.08 |  | 0.21 |
| 3 | 1.37 | † | 0.22 |  | 1.08 |  | 0.20 |
| 4 (highest; ref) |  |  |  |  |  |  |  |
| Occupation |  |  |  |  |  |  |  |
| Managerial (ref) |  |  |  |  |  |  |  |
| Sales / Clerical | 0.77 | † | 0.12 |  | 0.95 |  | 0.18 |
| Service | 0.70 | † | 0.14 |  | 1.22 |  | 0.29 |
| Manual / Operators | 0.69 | * | 0.13 |  | 1.03 |  | 0.23 |
| *Geography* |  |  |  |  |  |  |  |
| Beale Rural - Urban code |  |  |  |  |  |  |  |
| Urban (ref) |  |  |  |  |  |  |  |
| Suburban | 0.95 |  | 0.13 |  | 1.12 |  | 0.18 |
| Rural | 1.03 |  | 0.14 |  | 0.92 |  | 0.15 |
| Region |  |  |  |  |  |  |  |
| Northeast (ref) |  |  |  |  |  |  |  |
| Midwest | 1.65 | ** | 0.31 |  | 1.09 |  | 0.23 |
| South | 1.63 | ** | 0.29 |  | 1.21 |  | 0.24 |
| West | 1.84 | ** | 0.36 |  | 1.91 | ** | 0.40 |
| *Health Conditions* |  |  |  |  |  |  |  |
| Depression | 0.93 |  | 0.11 |  | 1.06 |  | 0.15 |
| Back pain | 1.20 |  | 0.15 |  | 1.40 | * | 0.21 |
| Arthritis | 1.28 | † | 0.18 |  | 2.11 | *** | 0.37 |
| Pain interference |  |  |  |  |  |  |  |
| Non-interfering pain (ref) |  |  |  |  |  |  |  |
| Interfering pain | 1.21 |  | 0.15 |  | 1.58 | ** | 0.23 |
| ADL | 1.17 |  | 0.17 |  | 1.19 |  | 0.19 |
| IADL | 1.01 |  | 0.16 |  | 1.05 |  | 0.19 |
| Ever had cancer | 1.23 |  | 0.20 |  | 0.96 |  | 0.19 |
| Lung disease | 1.16 |  | 0.22 |  | 0.85 |  | 0.20 |
| Heart disease | 1.15 |  | 0.15 |  | 0.86 |  | 0.14 |
| Stroke | 1.33 |  | 0.28 |  | 1.26 |  | 0.32 |
| Diabetes | 0.84 |  | 0.12 |  | 0.86 |  | 0.14 |
| High blood pressure | 1.24 | † | 0.14 |  | 1.11 |  | 0.15 |
| *Healthcare related characteristics* |  |  |  |  |  |  |  |
| Care satisfaction |  |  |  |  |  |  |  |
| Neutral (ref) |  |  |  |  |  |  |  |
| Satisfied | 0.93 |  | 0.11 |  | 0.96 |  | 0.13 |
| Dissatisfied | 0.81 |  | 0.16 |  | 1.14 |  | 0.25 |
| # Times seen a doctor |  |  |  |  |  |  |  |
| 0-4 times (ref) |  |  |  |  |  |  |  |
| 5-10 times | 1.23 |  | 0.18 |  | 1.07 |  | 0.19 |
| 11+ times | 1.40 | * | 0.21 |  | 1.80 | ** | 0.31 |
| Insurance status |  |  |  |  |  |  |  |
| Uninsured (ref) |  |  |  |  |  |  |  |
| Any private | 1.35 |  | 0.31 |  | 1.65 | † | 0.44 |
| Public only | 1.33 |  | 0.32 |  | 1.60 | † | 0.44 |
| *Constant* | 0.05 | *** | 0.02 |  | 0.01 | *** | 0.01 |

*Note.* ADL = activities of daily living; IADL = instrumental activities of daily living. Base outcome is no reported opioid use.

† p<.10 * p<.05; ** p<.01; *** p<.001.
